# The use of technologies and social media in patients with schizophrenia and schizoaffective disorder

**DOI:** 10.1192/j.eurpsy.2022.2009

**Published:** 2022-09-01

**Authors:** O. Elleuch, L. Zouari, S. Omri, R. Feki, I. Gassara, N. Smaoui, J. Ben Thabet, N. Charfi, M. Maalej, M. Maalej

**Affiliations:** Hedi Chaker University Hospital, Psychiatry, Sfax, Tunisia

**Keywords:** Schizoaffective disorder, schizophrénia, technologies, social media

## Abstract

**Introduction:**

Technologies such as the phone , the computer , and social media network nowadays are becoming more and more available to everyone including patients with mental illnesses.

**Objectives:**

Our study aimed to examine the prevalence of technology use in individuals with schizophrenia and schizoaffective disorder.

**Methods:**

Study participants were recruited from the outpatient unit of the department C of psychiatry in Hedi Chaker hospital of Sfax , Tunisia. A total of 38 male patients were recruited , from whom the diagnosis of schizophrenia or schizoaffective disorder according to the DSM-5 criteria had been confirmed. Socio-demographic and clinical information as well as details about their technology use were was collected from all the patients.

**Results:**

Of the 38 study participants, 65.8% owned a cell phone , and 52.6% used the cell phone to send or receive messages. A rate of 21.1% owned a computer , 34.2% had internet access and 28.9% had an email account. A rate of 23.7% used social media. Facebook was the most popular social media site. 72% of cell phone owners would like to communicate with their doctor via text messages , and 68% would like to be reminded of their appointments via text messages. Among social media users , 55.6% expressed their interest in a social-media-based doctor-patient communication and appointment reminders.

**Conclusions:**

Our findings suggest that these technologies afford an opportunity to improve the management of these patients.

**Disclosure:**

No significant relationships.

